# Study of the brain function characteristics in children with cerebral palsy during walking using functional near-infrared spectroscopy

**DOI:** 10.1117/1.NPh.12.2.025004

**Published:** 2025-03-31

**Authors:** Tengyu Zhang, Gongcheng Xu, Yajie Chang, Zichao Nie, Aiping Sun, Zengyong Li, Ping Xie

**Affiliations:** a National Research Center for Rehabilitation Technical Aids, Beijing Key Laboratory of Rehabilitation Technical Aids for Old-Age Disability, Key Laboratory of Neuro-functional Information and Rehabilitation Engineering of the Ministry of Civil Affairs, Beijing, China; b The Hong Kong Polytechnic University, Faculty of Engineering, Department of Biomedical Engineering, Hong Kong, China; c Yanshan University, Institute of Electric Engineering, Key Laboratory of Intelligent Rehabilitation and Neuromodulation of Hebei Province, Qinhuangdao, China

**Keywords:** cerebral palsy, functional near-infrared spectroscopy, cortical activation, functional connectivity, information flow

## Abstract

**Significance:**

Abnormal gait of children with cerebral palsy (CP) is caused by brain damage or developmental defects, exploring the brain’s functional characteristics and regulatory mechanisms is essential for rehabilitation.

**Aim:**

We aim to study the brain function characteristics in children with CP during walking.

**Approach:**

The cortical activation, functional connectivity, information flow, and dynamic state transitions of 17 children with CP and 13 healthy children (HC) were analyzed in the resting and walking states.

**Results:**

The motor cortex (MC) of HC is significantly activated in the walking state, whereas both the prefrontal cortex (PFC) and MC of children with CP are significantly activated. The resting brain functional connectivity of children with CP decreased and showed higher global efficiency and modularity and lower clustering coefficients and local efficiency. During walking, the brain network of children with CP was difficult to maintain a stable global high-connectivity state so the local high-connectivity state became the main connectivity state. For children with CP, more brain resources were allocated to the non-dominant MC during walking, whereas more brain resources were allocated to the dominant MC in HC.

**Conclusions:**

These indicators reflect the characteristics of brain activation, network connectivity, and information regulation in children with CP, which provide the theoretical basis for targeted rehabilitation treatment.

## 1 Introduction

Cerebral palsy (CP) is a nonprogressive neurological disorder caused by damage to the developing brain and is often accompanied by sensory and perceptual deficits.^1^ Spasticity is the most common type of CP and is characterized by increased muscle tone and stiffness.^2^ Children with spastic CP exhibit significantly abnormal gait patterns due to upper neuron damage, and improving gait has always been a challenge in their rehabilitation treatment.^3^^,^^4^ Currently, gait interventions for spastic CP primarily focus on physical stimulation and gait training, which have shown some effectiveness.^5^^,^^6^ However, human movement control involves the coordination of various neural, motor, and sensory functions, as well as information interactions.^7^ The fundamental cause of motor and postural abnormalities in children with CP is damage to or developmental defects in neural circuits, leading to abnormal muscle coordination.^8^^,^^9^ The rehabilitation process requires central-peripheral cooperative intervention to promote the reconstruction of neural circuits and the reorganization of functional networks.^10^^,^^11^ Therefore, investigating the brain network characteristics and regulatory mechanisms in children with CP while walking is essential for central intervention and functional reconstruction.

Functional magnetic resonance imaging (fMRI) has been an effective tool for early research on the brain function and pathogenesis because of its advantages of being noninvasive and capable of multiplanar imaging.^12^^,^^13^ To date, fMRI technology has made significant contributions to the in-depth study of neural plasticity following motor intervention training^14^ and abnormal brain structure and gait balance^15^^,^^16^ in children with CP. Simultaneously, electroencephalography (EEG), a commonly used portable tool for studying brain function, has played a vital role in research on sensory-motor processing,^17^ brain function assessment,^18^ and disease prediction^19^ in children with CP. However, these techniques are difficult to use for detecting brain function in children with CP while walking because of limitations in the testing environment and susceptibility to motion interference. Functional near-infrared spectroscopy (fNIRS) is an efficient optical neuroimaging technique that uses the characteristics of near-infrared spectroscopy to detect changes in oxyhemoglobin ($\Delta {\mathrm{HbO}}_{2}$) and deoxyhemoglobin (ΔHbR) concentrations in brain regions and assesses brain function based on these changes.^20^ Compared with traditional brain function detection methods such as MRI and EEG, fNIRS results in greater resistance to motion and electromagnetic interference, making it suitable for studying movement tasks.^21^^–^^24^ Researchers have utilized fNIRS to observe cortical activation in children with CP during movement tasks.^25^ In our previous study, we analyzed the characteristics of brain network connectivity in children with CP during upper and lower limb movement training tasks and proposed several near-infrared indicators for the assessment of motor function.^26^^,^^27^ Considering the nonstationary and multiscale characteristics of fNIRS signals,^28^^,^^29^ the wavelet transform can be used to analyze fNIRS signals from different perspectives in the time and frequency domains.^30^^,^^31^ Therefore, this study employed fNIRS technology and the Molet continuous wavelet transform analysis method to comprehensively investigate the brain functional characteristics of children with CP while walking using indicators such as wavelet amplitude, wavelet coherence, and phase transfer entropy. In addition, dynamic brain functional network analysis was conducted to explore the dynamic changes in brain network connectivity during walking in children with CP. These studies provide a theoretical basis for further central nervous system intervention and functional assessment.

## 2 Methods

### 2.1 Participants

Ethical approval for this study was obtained from the Ethics Committee of the Rehabilitation Hospital Affiliated with the National Research Center for Rehabilitation Aids in China. Considering that brain lesions tend to stabilize in children after the age of 3,^32^ 17 children with spastic CP aged over 3 years were recruited from the hospital. The inclusion criteria for patients were as follows: (1) aged between 3 and 14 years, (2) able to understand the experimental requirements and cooperate in completing the tasks, (3) no other diseases affecting motor function or brain function in addition to spastic CP, and (4) a GMFCS level of I or II for children with CP. In addition, 13 age-matched healthy children (HC) were recruited from the community as a control group. The inclusion criteria for the control group were as follows: (1) aged between 3 and 14 years, (2) no history of developmental delay and no other neurological diseases affecting walking, (3) no obvious abnormal posture in either lower limb, such as knock-knees or bowlegs, (4) no orthopedic surgery on either lower limb within the past 6 months, and (5) ability to cooperate in completing the experiment. All the information of the subjects was shown in Table 1. The guardians of the children were informed of the experimental procedures and content and signed informed consent forms.

**Table 1:** Information of CP subjects.

Subjects	Gender	Age (years)	GMFCS	Dominant side
1	M	10	I	R
2	M	7	I	L
3	M	10	I	R
4	M	12	II	R
5	M	10	II	R
6	M	13	II	R
7	M	8	I	R
8	M	10	I	L
9	F	11	II	R
10	F	4	I	R
11	F	4	I	R
12	F	3	I	R
13	F	5	I	L
14	M	5	I	R
15	M	8	II	R
16	M	10	I	R
17	M	14	II	R
18	M	8	/	R
19	F	13	/	R
20	F	13	/	R
21	F	6	/	R
22	M	6	/	R
23	F	5	/	R
24	F	12	/	R
25	F	8	/	R
26	F	11	/	R
27	M	3	/	R
28	F	12	/	R
29	M	8	/	R
30	M	7	/	R

*Note*: Subjects 1 to 17 were in the CP group and subjects 18 to 30 were in the HC group. The difference in age between the two groups was not statistically significant ($p_{\left(t\text{-}\text{test}\right)}=0.9064$, t=−0.1186).

### 2.2 Procedures

**Fig. 1:**
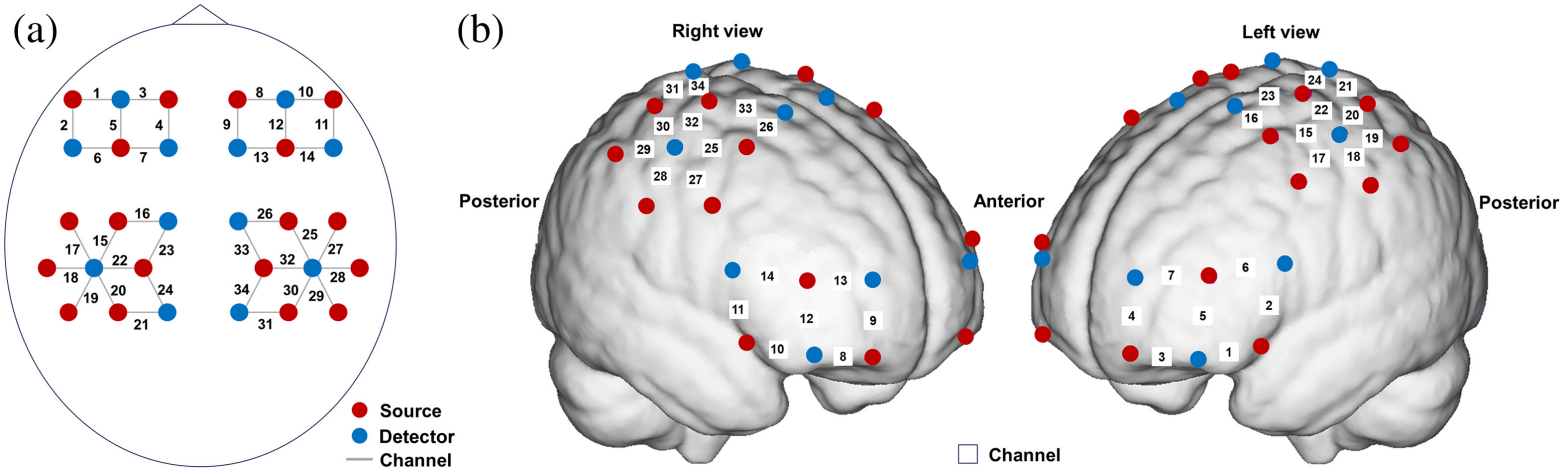
Configuration of light sources, detectors, and measurement channels: (a) the optodes design. The red dots represent the light sources, and the blue dots represent the detectors. Each pair of a light source and a detector forms a channel. A total of 20 light sources and 16 detectors were used, forming 34 channels. (b) The corresponding location of optodes and channels on the surface of the brain, primarily covering the prefrontal lobe and motor areas.

The participants’ $\Delta {\mathrm{HbO}}_{2}$ and ΔHbR signals from the prefrontal cortex (PFC) and motor cortex (MC) in both hemispheres were collected during the resting and free walking states using the NirSmartII-3000A equipment (Danyang Huichuang Medical Equipment Co., Ltd., China). The system consists of a near-infrared light source [light-emitting diodes (LED)] and an avalanche photodiode (APD) as detectors, with wavelengths of 730 and 850 nm, respectively. Twelve detectors and 18 light sources were affixed to the head cap, forming a total of 34 channels (Fig. 1), with 3 cm between each detector and light source. During cap fitting, the nasion, inion, and left/right preauricular points were used as anatomical reference points to ensure accurate channel positioning. The data acquisition frequency was set at 10 Hz.

During the experiment, the participants were seated in a quiet environment for 5 min to relax. Subsequently, they were instructed to maintain a comfortable sitting posture with their eyes closed for 6 min in the resting state. Then, they were asked to walk back and forth on a 10-m walkway at their most comfortable pace. Each trial began with 10 s of standing, followed by 6 min of walking and another 10 s of standing before sitting down. During the standing periods, the participants were asked to gaze forward and avoid thinking about anything specific.

### 2.3 Data Processing and Analysis

#### 2.3.1 fNIRS data preprocessing

The fNIRS data were preprocessed using MATLAB software. Channels with an optical intensity greater than 1000 or less than 0.5 and channels with a quotient of mean and standard deviation less than 2 were excluded,^33^^–^^35^ these excluded channels were no longer involved in average processing and statistical analysis. The recorded optical intensity data were first converted to optical density data using the hmrIntensity2OD function in the Homer2 toolbox, and baseline drift and peak artifacts were eliminated using the temporal derivative distribution repair (TDDR) algorithm.^36^^,^^37^ To address potential spikes introduced by the TDDR algorithm, the Hampel filtering was applied to the decomposed high-frequency components during the implementation of the TDDR algorithm (using the Hampel function in MATLAB, with the number of data points on each side set to 50 and the standard deviation multiplier set to 4).^34^ Afterward, the modified Lambert-Beer law was used to convert to $\Delta {\mathrm{HbO}}_{2}$ and ΔHbR, and the differential path length factor values were calculated based on the age of the subjects,^38^ whereas the second-order detrending method was used to eliminate signal drift.^39^ Finally, systematic physiological contamination was mitigated using principal component analysis to remove the first component corresponding to the largest eigenvector.^40^^,^^41^

Considering that the present study needed to explore the intensity of cortical activity of resting-state cortical hemoglobin signals, as well as task-state brain functional activity and network metrics, the analysis was focused on 0.01 to 0.2 Hz.^39^^,^^42^^,^^43^ A continuous wavelet transform was employed to identify and retain 0.01 to 0.2 Hz oscillation signals as neurophysiological hemodynamic responses.^44^ The Morlet wavelet, known for its excellent localization properties in both the time and frequency domains, was chosen as the mother wavelet. The definition of the wavelet transform is as follows:

$$W(s,t)=\frac{1}{\sqrt{s}}\int_{-\infty }^{+\infty }\varphi (\frac{u-t}{s})g(u)du,$$ (1)

$$\varphi (u)=\frac{1}{\sqrt[{4}]{\pi }}\cdot e^{-i\omega_{0}u}\cdot e^{-\frac{u^{2}}{2}},$$ (2)

where φ(u) is the mother wavelet, g(u) is the original time series, and W(s,t) is the complex wavelet coefficient.

In the data analysis process, the PFC and MC from the dominant hemisphere (ipsilateral hemisphere to the affected side) were marked as DPFC and DMC, and PFC and MC from the nondominant hemisphere (contralateral hemisphere to the affected side) were marked as NPFC and NMC.

#### 2.3.2 Static brain function analysis based on the wavelet transform

##### Cortical response intensity and lateralization

The wavelet amplitude (WA) calculated by the wavelet transform reflects the fluctuation amplitude of the signal at a certain frequency, which can be used as an indicator of the activation of cortical regions over time. Higher WA values often indicate stronger hemodynamic responses.^45^^,^^46^ The hemodynamic response intensity of a brain region is calculated as the average WA value of all channels in that brain region. In addition, the lateralization index (LI) is used to quantify the balance of hemodynamic responses between cerebral hemispheres.^47^ A LI value of 1 indicates complete ipsilateral activation, whereas a value of −1 indicates complete contralateral activation. The specific calculation is as follows:

$$WA(i)=\frac{1}{t(s_{2}-s_{1})}\int_{0}^{t}\int_{s_{1}}^{s_{2}}|W(s,t)|ds\;dt,$$ (3)

$$LI=\frac{WA_{i}-WA_{o}}{WA_{i}+WA_{o}},$$ (4)

where WA(i) represents the wavelet amplitude of channel i, $s_{2}$ and $s_{1}$ represent the end and start scales at 0.01−0.2  Hz, respectively. The mean wavelet amplitude value of the ipsilateral brain channels is represented by $WA_{i}$, whereas the mean wavelet amplitude value of the contralateral brain channels is represented by $WA_{o}$.

##### Functional connectivity analysis based on wavelet coherence

Functional connectivity is primarily measured by the synchrony or correlation between the time series of nodes in brain regions.^48^ Wavelet coherence (WCO) can be used to quantify the correlation of signals at multiple scales within a fixed frequency band.^49^^,^^50^ Given two time series x(t) and y(t), the wavelet power and WCO at different scales corresponding to frequencies were calculated as follows:

$$P_{K}(f)=\frac{1}{N}\overset{N}{\underset{n=1}{\sum }}\omega_{k}(t_{n})w_{k}^{*}(t_{n})=\frac{1}{N}\overset{N}{\underset{n=1}{\sum }}w_{k}^{2}(f,t_{n}),$$ (5)

$$\mathrm{WCO}(f)=\frac{\left[\frac{1}{N}\sum_{n=1}^{N}\omega_{x}(t_{n})w_{y}^{*}(t_{n})][\frac{1}{N}\sum_{n=1}^{N}\omega_{y}(t_{n})w_{x}^{*}(t_{n})\right]}{P_{x}(f)P_{y}(f)},$$ (6)

where $\omega_{k}(t_{n})$ represents the transformed complex time series, which is calculated as follows:

$$\omega_{k}(t_{n})=a_{k}(f,t_{n})+ib_{k}(f,t_{n}),$$ (7)

$$W_{K}(f,t_{n})=\sqrt{a_{k}^{2}(f,t_{n})+b_{k}^{2}(f,t_{n})}.$$ (8)

The average WCO values at multiple frequencies corresponding to the 0.01 to 0.2 Hz frequency band were calculated as indicators of functional connectivity. A higher WCO value indicates a stronger linear relationship between signals, implying stronger functional connectivity. Functional connectivity between brain regions was revealed as the average WCO value of all channel pairs across the brain regions.

To further evaluate the topological structure of the brain network constructed by WCO and the information transmission between brain nodes, clustering coefficients (CC), global efficiency (GE), local efficiency (LE), and modularity were calculated at 0.1 to 0.5 sparsity levels using graph theory methods.^51^^,^^52^ The CC is a measure of network segregation, GE is used to measure network integration, and LE measures the integration ability between neighboring nodes of a given node. The equations for these parameters are as follows:

$$CC=\frac{1}{m}\overset{m}{\underset{i=1}{\sum }}C_{i}=\frac{1}{m}\overset{m}{\underset{i=1}{\sum }}\frac{E_{i}}{k_{i}(k_{i}-1)/2},$$ (9)

where $C_{i}$ represents the CC of node i, $E_{i}$ represents the number of neighbor nodes directly connected to node i, $k_{i}$ represents the degree of node i, and n is the total number of nodes.^52^

$$\mathrm{GE}=\frac{1}{n}\overset{n}{\underset{i=1}{\sum }}E_{i}=\frac{1}{n}\overset{n}{\underset{i=1}{\sum }}\frac{\overset{n}{\underset{j=1,j\neq i}{\sum }}d_{ij}^{-1}}{n-1},$$ (10)

where $E_{i}$ represents the GE of node i, $d_{ij}$ represents the shortest path length between node i and node j, and n is the total number of nodes.^52^

The LE of each node can be calculated as the global efficiency of that node’s neighborhood subgraph $G_{i}$. The local efficiencies of all nodes within the network are further averaged to estimate the network LE, which is calculated as follows:^53^

$$\mathrm{LE}=\frac{1}{n}\underset{i\in n}{\sum }\mathrm{GE}(G_{i}).$$ (11)

The calculation equation for modularity is

$$\text{modularity}(p)=\overset{m-1}{\underset{M}{\sum }}[\frac{l_{m}}{L}-(\frac{d_{m}}{2L}{)}^{2}],$$ (12)

where L is the number of connections in the network, $l_{m}$ is the number of connections between the nodes in module m, and $d_{m}$ is the sum of the degrees of the nodes in module m.^54^

To eliminate the influence of different sparsity levels on the results, the area under the curve (AUC) of the graph theory parameters was calculated to reflect the density of the brain network and the efficiency of information transfer.

##### Effective network analysis based on phase information

Effective networks can be used to analyze the dynamic interactions among brain regions, and a common method with which to evaluate effective networks is transfer entropy.^55^ Phase transfer entropy (PET) is a form of transfer entropy applied to the phase time series of signals. It is a causality-based nonlinear information-theoretical analysis method that can assess the direction of information transmission between two time series in real time, with advantages in accuracy, interpretability, and processing of asymmetric data.^56^

For the source signals X(t) and Y(t), the instantaneous phase information at a certain frequency after being transformed by the Molet wavelet is defined as

$$\theta_{k}(f,t)=\arctan[b_{k}(f,t)/a_{k}(f,t)/].$$ (13)

The average phase information in the 0.01 to 0.2 Hz frequency band is further calculated to obtain the instantaneous phase $\theta_{x}(t)$ and $\theta_{y}(t)$ of the source signal. The phase transfer entropy from signal X(t) to Y(t) is defined as

$${\mathrm{PTE}}_{xy}=H(\theta_{y}(t+\delta ),\theta_{y}(t))+H(\theta_{y}(t),\theta_{x}(t))-H(\theta_{y}(t))-H(\theta_{y}(t+\delta ),\theta_{y}(t),\theta_{x}(t))\;=\sum p(\theta_{y}(t+\delta ),\theta_{y}(t),\theta_{x}(t))\log(\frac{p(\theta_{y}(t+\delta )|\theta_{y}(t),\theta_{x}(t))}{p(\theta_{y}(t+\delta )|\theta_{y}(t))}),$$ (14)

where $\theta_{y}(t+\delta )$ represents the instantaneous phase time series of Y(t) at a certain delay. The probability of the data is calculated using the histogram method, with the width of the histogram defined according to Scott’s choice:

$$\text{binsize}=3.49\times \text{mean}(\mathrm{std}(\theta ))\times Ns^{-\frac{1}{3}},$$ (15)

where Ns represents the sample size and θ represents the instantaneous phase matrix of the signal. The delay time is calculated using the phase reversal method.^57^

Because PTE lacks a meaningful upper limit, it is standardized to eliminate bias and better represent the differences in information intensity between brain regions

$$d{\mathrm{PTE}}_{xy}=\frac{{\mathrm{PTE}}_{xy}}{{\mathrm{TE}}_{xy}+{\mathrm{PTE}}_{yx}}.$$ (16)

The range of dPTE is between 0 and 1. Within this range, if 0.5<dPTE<1, information flow is prioritized from X to Y. Conversely, if 0<dPTE<0.5, information flow is prioritized from Y to X. When dPTE equals 0.5, no preferred direction of information flow exists.^58^ Based on the strength and direction of the directed network, the out-degree (the number of information flows) and the strength of information flow for each channel and brain region were statistically analyzed^59^ for subsequent quantitative analysis.

#### 2.3.3 Dynamic functional connectivity state analysis based on deep clustering

**Fig. 2:**
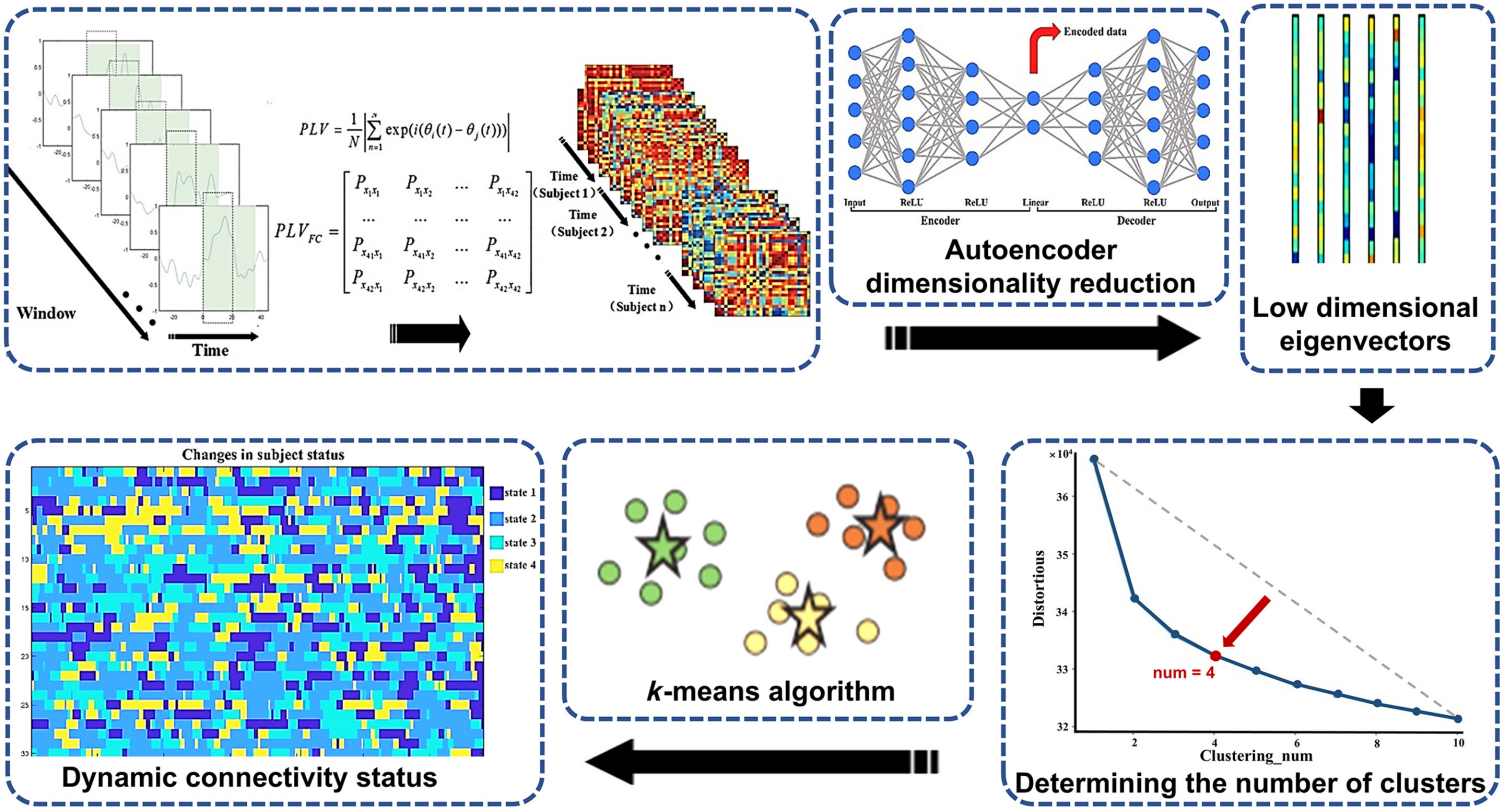
Dynamic functional connectivity analysis workflow. First, the sliding window method is used to divide the original data into several segments, and the PLV matrix for each sliding window is calculated as the feature vector. Subsequently, dimensionality reduction is performed on the feature vectors, and K-means clustering analysis is applied. The elbow method is used to determine the optimal number of states. Finally, the dynamic connectivity states of the brain are analyzed.

To further investigate the dynamic changes in functional connectivity states in MCs during the walking process, the functional connectivity matrix was subjected to clustering analysis using the K-means method,^60^ which represents the functional connectivity states during different time periods. This was performed to compare the differences in functional connectivity state changes between the HC and CP groups. Phase synchrony is an important indicator for measuring functional connectivity.^61^^,^^62^ A functional connectivity matrix for 0.01 to 0.2 Hz blood oxygen signals was calculated using the phase locking value (PLV) based on the Hilbert transform^26^ to improve the computational efficiency of dynamic functional connectivity analysis. The data of the 6-min walking process were divided into 340 segments vis a highly overlapping rectangular sliding window with a width of 20 s and a step length of 1 s. The PLV matrix for each sliding window was calculated, and the upper triangular elements of each PLV matrix were extracted as feature vectors. Due to the large error of the K-means clustering algorithm in clustering high-dimensional data, a deep learning framework was utilized to design an autoencoder for dimensionality reduction of the feature vectors,^63^ reducing the original 190 features to 64 features. Clustering analysis was subsequently performed on the feature matrix, with the number of states ranging from 1 to 10. The optimal number of states was determined using the elbow method. The clustering centers were obtained by repeating the process of randomly initializing the clustering centers 500 times^64^ and were used to analyze the brain’s connection states. The specific process was shown in Fig. 2. Dynamic indicators for each participant, such as the state frequency, state transition probability, average state duration, and state transition percentage,^64^ were calculated to reflect the dynamic changes in brain function.

### 2.4 Statistical Analysis

IBM SPSS Statistics 27 was used for statistical analysis. Statistical tests were performed on the WA, LI, WCO, and information flow intensity of children with CP and HC in different states. First, the normality test and homogeneity of variance test were conducted. For data that met the criteria of normal distribution and homogeneity of variance, an independent samples t-test was used to compare the variables between the two groups. For data that did not meet the criteria, the Mann–Whitney U test was used. The statistical significance level was set at p=0.05. To control for false-positive rates, the Bonferroni correction was applied to the multiple comparisons. For the cortical activation, functional connectivity, and effective connectivity, four conditions were compared (HC_rest, HC_task, CP_rest, CP_task,) so the threshold of significance was adjusted to 0.0125 (0.05/4).

## 3 Results

The results reported in this section were all based on $\Delta {\mathrm{HbO}}_{2}$, and the results based on ΔHbR were presented in the Supplementary Materials.

### 3.1 Cortical Response Intensity and Lateralization

**Fig. 3:**
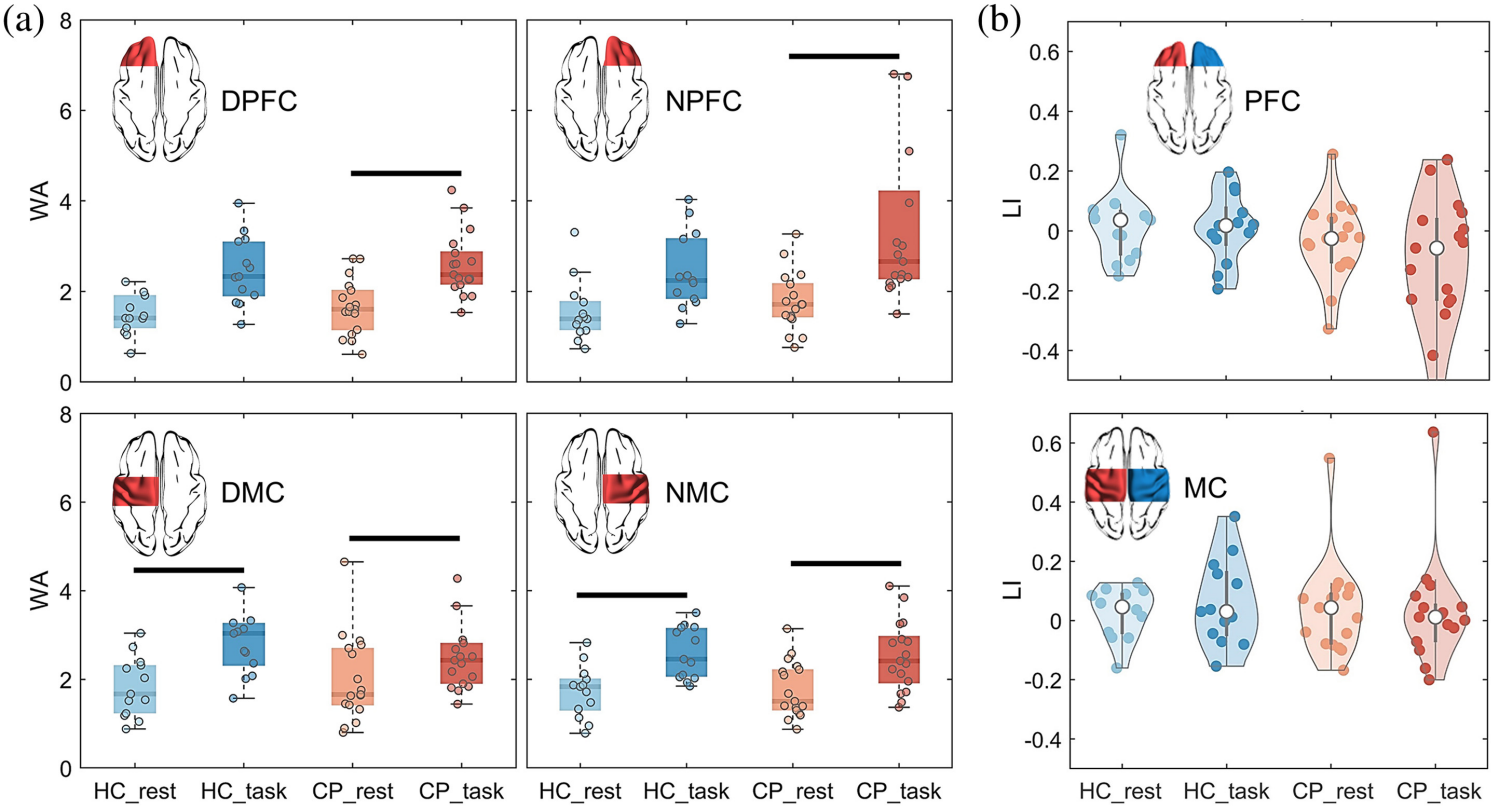
WA and LI results: (a) average WA values of the brain region. A horizontal black line indicates a significant difference (p<0.0125). Compared with the resting state, children with CP showed significantly increased WA in all brain regions during the walking state, whereas healthy children only showed significantly increased WA in MC. (b) LI values of the PFC and MC. No significant difference was found between CP and HC groups and between resting and walking states.

The WA and LI results for the HC and CP groups in the resting and task states are shown in Fig. 3. As shown in Figs. 3(a) and 3(b), there was no significant difference between CP and HC groups in activation and lateral deviation of each brain region during resting and walking state. Compared with the resting state, children with CP showed significantly increased activation in all brain regions during the walking state (P<0.0125), whereas healthy children only showed significantly increased activation in MC (P<0.0125).

### 3.2 Functional Connectivity Analysis

**Fig. 4:**
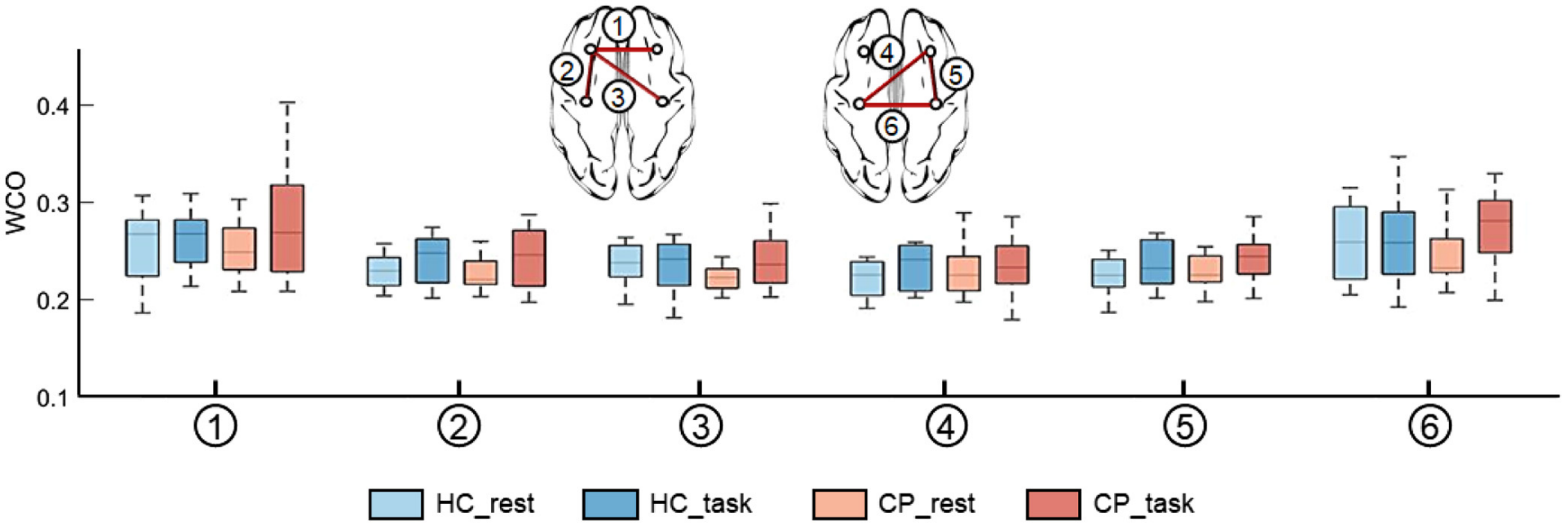
WCO between brain regions under different tasks. Numbers 1 to 6 represent different pairs of brain regions, in sequence as follows: DPFC-NPFC, DPFC-DMC, DPFC-NMC, NPFC-DMC, NPFC-NMC, and DMC-NMC. During the resting state, the WCO values of children with CP were all lower than those of HC. During the walking state, the WCO values between all brain regions showed obvious increases in children with CP, whereas only those of the DPFC-DMC and NPFC-DMC in the HC group obviously increased.

As shown in Fig. 4, compared with those of HC, the WCO values between all the brain regions in children with CP were lower during the resting state. However, the WCO values of NPFC-NMC and DMC-NMC in children with CP were higher than those of HC during the walking state. Compared with the resting state, in the walking state, only the WCO values of the DPFC-DMC and NPFC-DMC in the HC group showed obvious increases, whereas the WCO values between almost all brain regions in children with CP showed obvious increases.

**Fig. 5:**
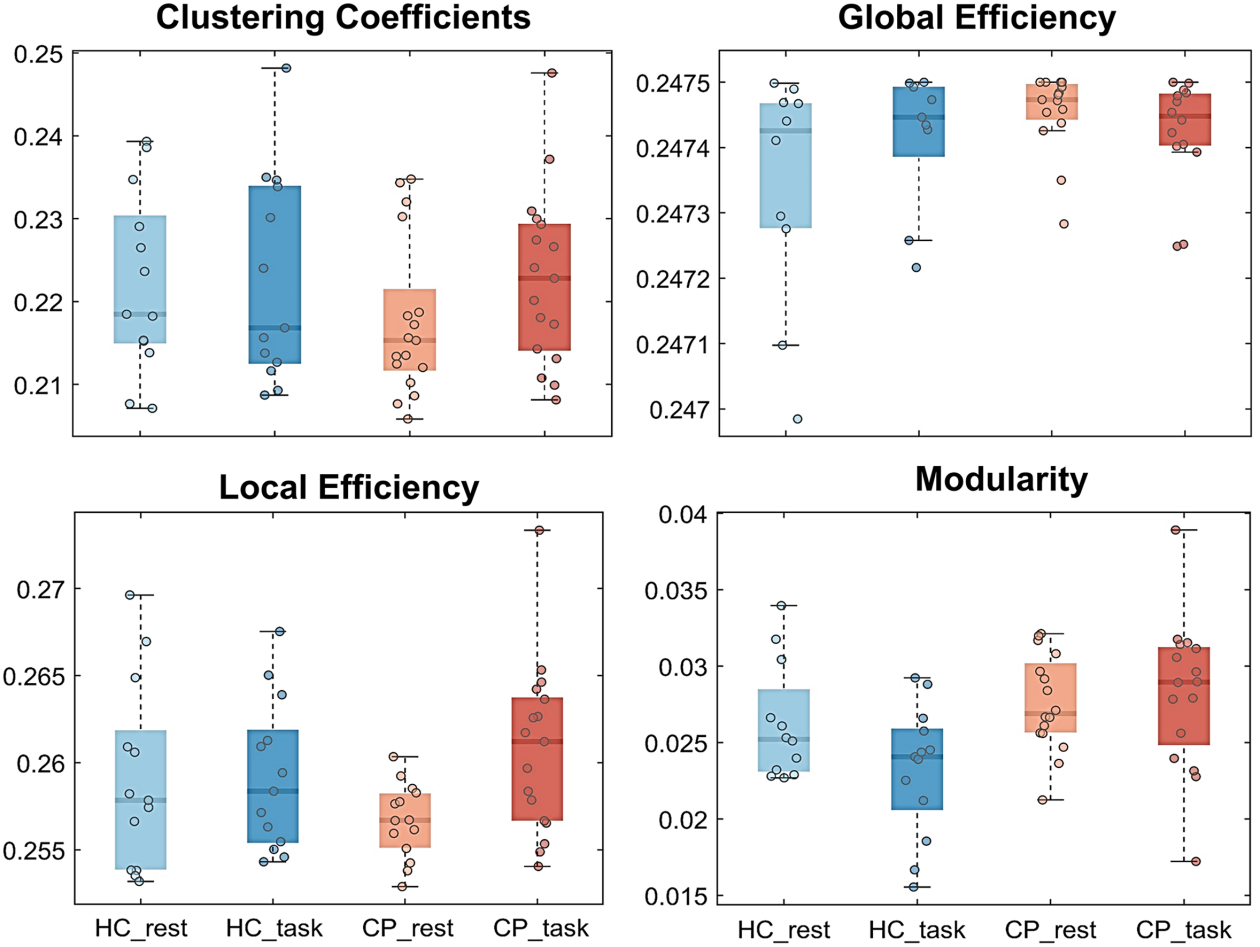
Results of graph theory analysis. The CC and LE of children with CP are lower than those of HC in the resting state and are greater than those of HC in the walking state. The GE and modularity of children with CP are greater than those of the HC.

As shown in Fig. 5, the CC and LE of children with CP are lower than those of HC in the resting state and are greater than those of HC in the walking state. The GE and modularity of children with CP are greater than those of the HC.

### 3.3 Effective Network Analysis

**Fig. 6:**
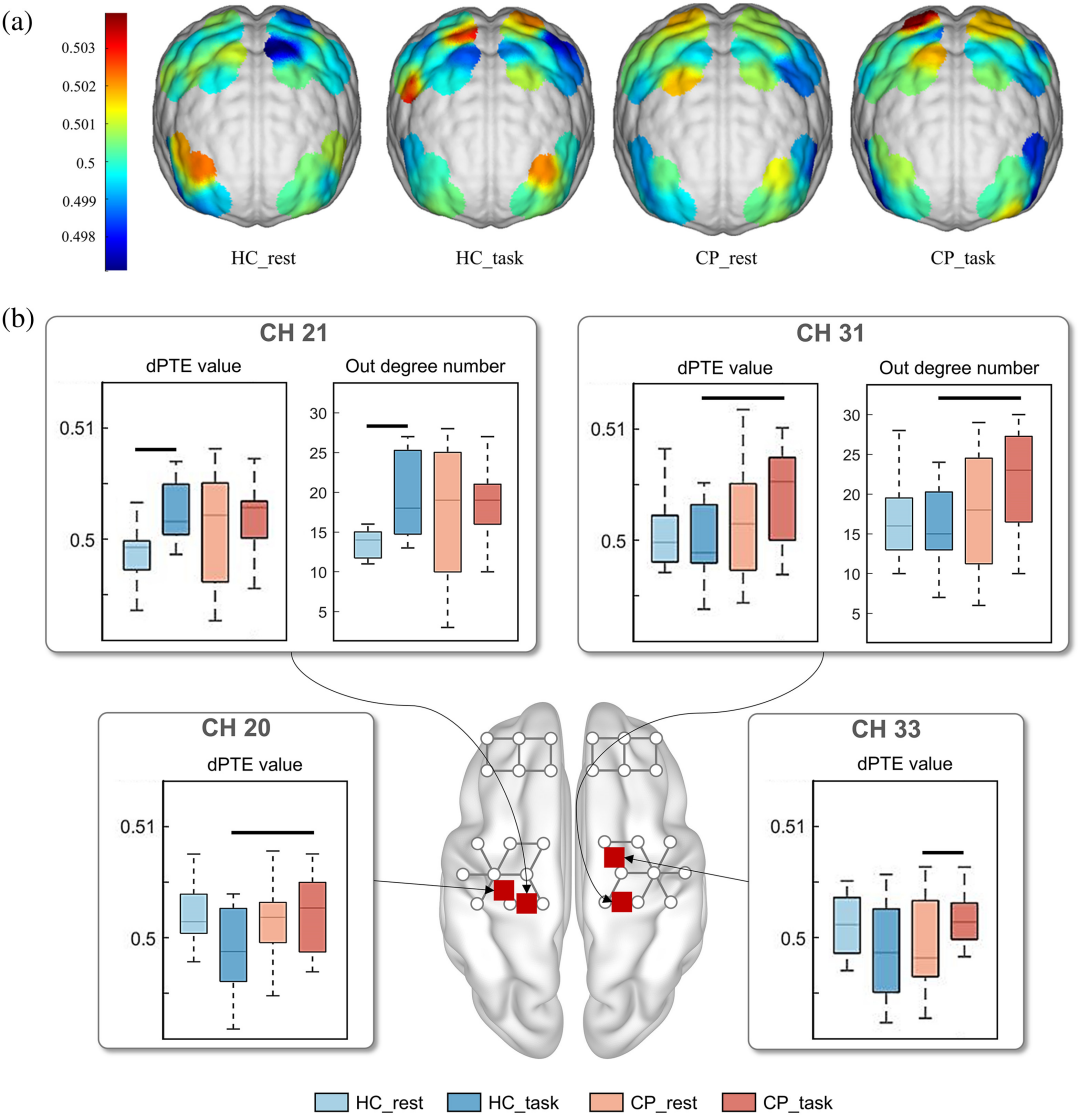
Results of effective network analysis: (a) average brain information outflow intensity. The information outflow from the PFC decreased during walking tasks, whereas the information outflow from the MC increased. The information flow in both sides of the MC increased in the walking state for the HC, whereas the NMC of the children with CP showed a greater increase. (b) Out-degree number and information outflow intensity of partial channels. A horizontal black line indicates a significant difference (p<0.0125). In the walking state, the out-degree of channel 31 and the information outflow intensity of channel 20 and channel 31 in the CP group were higher than those in the HC group. The out-degree and information outflow intensity of channel 21 in the HC group and information outflow intensity of channel 33 in the CP group were increased in the walking state.

The differences in the degree of brain information output and outflow intensity between the two groups of children were compared and analyzed under the resting and task states, and the results are shown in Fig. 6. Figure 6(a) reflects the situation of brain information outflow, indicating that the information outflow from the PFC decreased during walking tasks, whereas the information outflow from the MC increased. The difference between the two groups was that the information flow in both sides of the MC increased in the walking state for the HC, whereas the NMC of the children with CP showed a greater increase. Figure 6(b) shows that there was no significant difference in the out-degree and information outflow intensity between the two groups in the resting state. In the walking state, the out-degree of channel 31 in CP group was significantly higher than that in HC group (P=0.01), and the information outflow intensity of channel 20 (P=0.01) and channel 31 (P=0.007) in CP group was significantly higher than that in HC group. Compared with the resting state, the out-degree (P=0.004) and information outflow intensity (P=0.003) of channel 21 were significantly increased for the HC group in the walking state, and the information outflow intensity of channel 33 was significantly increased for the CP group in the walking state (P=0.012).

### 3.4 Dynamic Functional Connectivity State Analysis

**Fig. 7:**
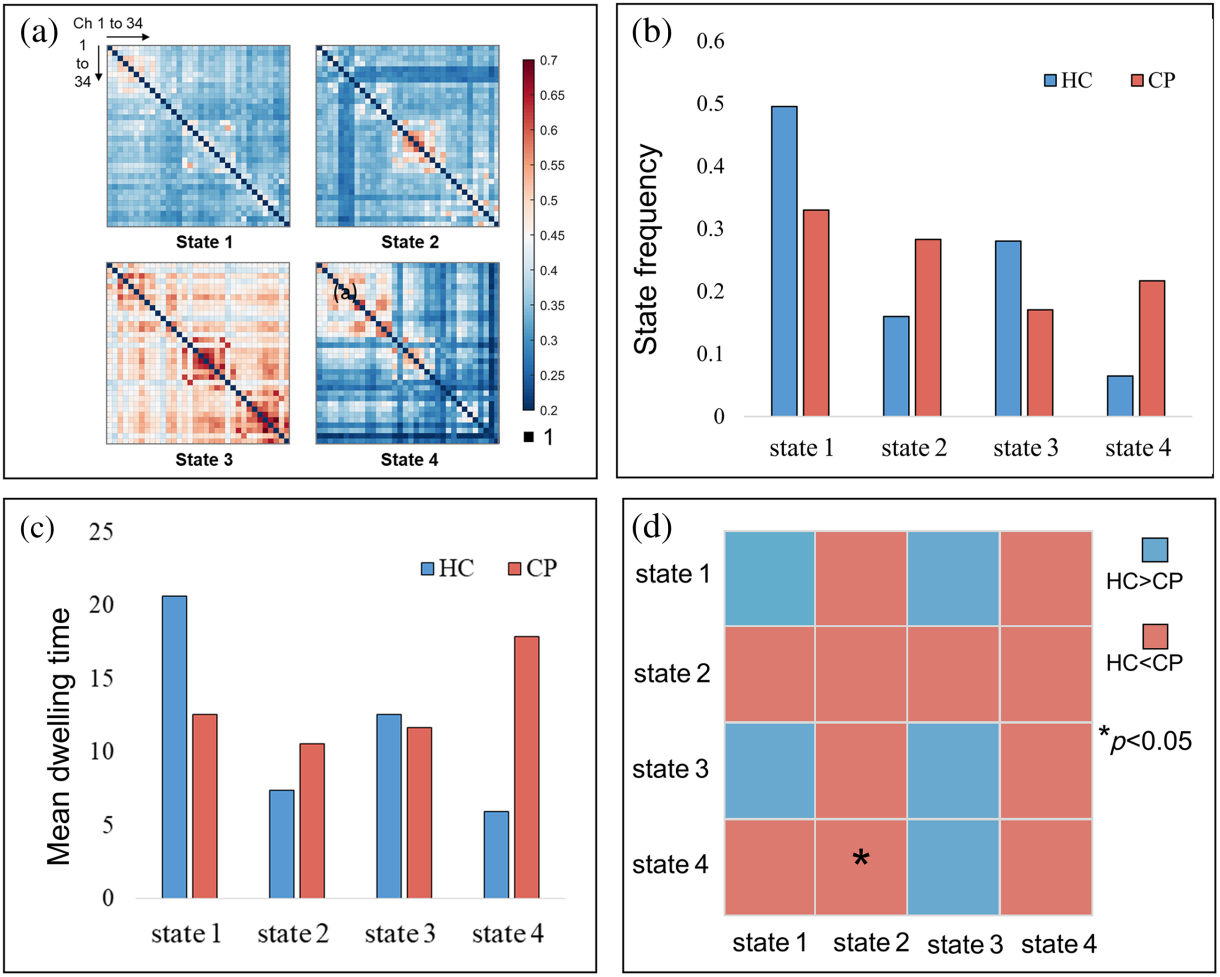
Results of dynamic brain network connectivity: (a) four states of functional connectivity. State 1 was a global low-connectivity state, state 3 was a global high-connectivity state, and states 2 and 4 were dominated by high-connectivity in local brain regions. State 2 mainly showed high connectivity in DMC and state 4 mainly showed high connectivity in PFC and DMC. (b) Frequency of state occurrence. The occurrence frequency of states 1 and 3 in children with CP was lower than those in the HC group, whereas the occurrence frequency of states 2 and 4 was the higher. (c) Average dwelling time. The CP group stayed shorter in states 1 and 3 and stayed longer in states 2 and 4. (d) Percentage matrix of state transition. The proportions of transitions from states 4 to 2 in children with CP were significantly greater than those in HC.

By conducting cluster analysis, four functional connectivity states were identified, and the results are shown in Fig. 7(a). State 1 was a global low-connectivity state, state 3 was a global high-connectivity state, and states 2 and 4 were dominated by high-connectivity in local brain regions. State 2 mainly showed high connectivity in DMC, and state 4 mainly showed high connectivity in PFC and DMC.

As shown in Fig. 7(b), during the walking process, compared with the HC group, the occurrence frequency of states 1 and 3 were lower in children with CP, whereas the occurrence frequency of states 2 and 4 were higher. Figure 7(c) shows that compared with the HC group, the CP group stayed shorter in states 1 and 3 and stayed longer in states 2 and 4. Figure 7(d) shows that the proportions of transitions from states 4 to 2 in children with CP were significantly greater than those in HC (p=0.018).

## 4 Discussion

Using fNIRS technology, this study analyzed the cortical activation and lateralization in children with CP while walking, calculated the functional connectivity and graph theory network parameters between brain regions, and used phase transfer entropy to measure the flow of information in the brain. Finally, dynamic state changes in functional connectivity in the MC were analyzed. The results revealed differences between children with CP and HC in terms of cortical activation, functional connectivity, and brain resource allocation.

### 4.1 Brain Activation Patterns

Higher WA values often indicate higher hemodynamic spectral power and are commonly used as indicators of activation during tasks. During the walking task, the activation of the PFC and MC in HC and children with CP increased to a certain extent compared with that in the resting state. The activation of the MC in HC significantly increased, whereas the activation of both PFC and MC in children with CP significantly increased. The regulation of the PFC and MC during walking in normal individuals involves complex neurophysiological processes.^55^ The PFC is primarily responsible for planning, decision-making, and motor control, whereas the MC is responsible for executing specific motor commands. Previous studies have also shown a correlation between somatosensory feedback and PFC activity during walking.^65^ Typically, walking requires the coordinated participation of cognitive and motor brain regions, leading to increased energy consumption in the relevant cortical areas. Therefore, the activation of the PFC and MC increased during motor tasks, which is consistent with previous findings on bilateral upper limb movement conditions.^66^ For HC, walking is a simple periodic rhythmic movement, and the execution of the movement task itself is not difficult. However, children with CP have certain impairments in motor control^66^ and sensory processing,^67^ and their abnormal gait patterns result in poor walking stability. They need to concentrate their attention to maintain balance while walking. Therefore, for children with CP, the walking task involves high-level cognitive tasks. This may explain why the activation of PFC in children with CP significantly increased.

### 4.2 Functional Connectivity

Functional connectivity based on WCO reflects the degree of correlation between brain regions, and by combining WCO values at different frequency scales, it can reveal the intrinsic connections of the brain within a fixed frequency band. In the resting states, the WCO values of each brain region in children with CP were lower than those in HC. This suggested a decrease in the number of local connections within the brain network, reflecting potential damage to the neural nodes and fiber bundles responsible for local information transmission,^68^ which results in sparse local connections in the brain network. This finding was consistent with previous studies on the functional connectivity of children with CP based on phase synchronization.^26^ Some studies based on DTI and fMRI have also shown that abnormal white and gray matter damage in the brains of children with spastic CP^69^ leads to a reduction in neural connectivity between brain regions.^70^ For HC, walking does not include much cognitive behavior, so only the functional connectivity between the dominant MC and PFC increased obviously during walking. However, in children with CP, the functional connectivity among brain regions was increased to a certain extent because both the PFC and MC are required to participate in the walking task. Although differences in functional connectivity were found between the two groups, no significant results were found, which may be due to the relatively good motor function (GMFCS I or II) of the CP selected in this study.

In addition, the graph theory analysis found that CC and LE of the brain network in children with CP were lower than those in HC during the resting state, and the GE and modularity were higher than those in HC. A study using white matter tractography also revealed a significant decrease in the CC of the children with CP,^71^ and a study about the functional network in early childhood found that the LE of young children and adolescents was significantly lower than that of adults.^54^ These findings demonstrated that functional segregation remained relatively steady, the brain possesses no optimal network configuration in the developmental periods of children, and the LE of the brain network will gradually increase with development. The LE of children with CP was lower than that of HC, indicating that their brain function development has been damaged or blocked. Some studies^72^^,^^73^ also found that damage to brain regions has a strong impact on local and global information transfer. The network architecture with less LE but high GE was more random and seemed to promote the re-learning of sensorimotor skills, but the performance is often less stable, even in well-recovered patients,^72^ which was consistent with the results of this study.

### 4.3 Brain Resource Allocation Patterns

In this study, information outflow in the brain was investigated via phase transfer entropy, which was based on wavelet transform. The results revealed that during the walking task, there was a decrease in information outflow from the PFC and an increase in information outflow from the MC. This is because walking in the laboratory is a task that does not involve complex cognitive behavior, the brain resources are mainly allocated to the MC for task execution. However, unlike the HC, the increase in information flow in children with CP was mainly in the MC of the non-dominant side, whereas the increase in HC was mainly in the MC of the dominant side. This may be because HC performs motor tasks mainly by the DMC, whereas children with CP perform the same task that is more difficult for the injured brain region, and the NMC needs to mobilize more brain resources to complete the task, resulting in an increase in information outflow. The above factors may explain the different patterns of information outflow between children with CP and HC, reflecting a special resource allocation pattern generated by the brain in children with CP to compensate for functional damage during the execution of motor tasks.^74^^,^^75^

### 4.4 Dynamic Functional Connectivity States during Walking

The results of dynamic functional connectivity analysis based on K-means indicate that the functional connectivity between brain regions is a constantly switching process during walking. For HC, brain network connectivity tends to be global high-connectivity and low-connectivity states, whereas the brain network connectivity of children with CP tends to be local high-connectivity and low-connectivity states. For example, the high-connectivity state of HC is mainly state 3, whereas the high-connectivity state of CP is mainly state 4. Previous research has found that brain lesions do not globally reduce connectivity in all functional systems of the brain but specifically alter connectivity of areas connected to that lesion.^72^ Therefore, the functional connectivity between the NMC and other brain regions in children with CP is greatly affected, as shown in state 4. The significantly higher switching frequency of children with CP from states 4 to 2 than that of HC may be mainly due to the higher proportion of states 4 and 2 in children with CP. When damage to a brain region leads to unstable connectivity, information transfer is achieved by increasing the frequency and dwelling time of connectivity between other brain regions. This may constitute a compensatory regulatory strategy generated by children with CP to compensate for functional brain damage.

## 5 Limitations

Children aged 3 to 14 years were selected as subjects in this study. They are in the stage of brain development, and their cortical responses and network connectivity patterns may be affected by age, which is one of the limitations of this study. However, many previous studies on brain function in children with CP^26^^,^^27^^,^^76^ also selected subjects with a wide age range and revealed abnormal changes in brain function. In future research, we will investigate specific brain development theories at different age stages and further classify children with spastic CP of different types and ages for more detailed research. In addition, because the equipment used in this study did not utilize short channels, we employed the PCA filtering method to minimize scalp surface noise interference as much as possible.

## 6 Conclusion

With fNIRS technology, wavelet transform and sliding window dynamic analysis were utilized to study cortical activation and brain network connectivity characteristics in children with CP during walking tasks in this study. Regarding cortical activation, compared with the resting state, the MC of HC was significantly activated in the walking state, whereas both the PFC and MC of children with CP were significantly activated due to the participation of cognitive tasks. Due to local brain injury or dysplasia, the resting brain functional connectivity of children with CP decreased, and it was difficult to maintain a stable global high-connectivity state during walking, then the local high-connectivity state became the main connectivity state. Accordingly, the network topology parameters showed higher GE and modularity and lower CC and LE in children with CP. This brain network has a tendency of randomization and tends to be unstable but is beneficial to promote motor relearning ability. The results of the information flow reflected that for children with CP, more brain resources were allocated to the NMC during walking, whereas more brain resources were allocated to the DMC in HC. These findings reflected a change in the brain’s regulatory strategy for children with CP to perform walking tasks.

The indicators studied in this paper, from the perspectives of activation, functional connectivity, information flow, and dynamic functional connectivity, reflected the information transmission, interaction, and brain regulation of the brain network in children with CP during walking tasks, which can provide a solid foundation for guiding clinical functional assessment and the formulation of rehabilitation strategies.

## Supplementary Material

10.1117/1.NPh.12.2.025004.s01

## Data Availability

The data that support the findings of this article are not publicly available due to privacy. They can be requested from the author at zhangtengyu1985@163.com.
