# Supplementary material for: Study of the brain function characteristics in children with cerebral palsy during walking using functional near-infrared spectroscopy
Source: Neurophotonics. 2025 Mar 31;12(2):025004. doi: 10.1117/1.NPh.12.2.025004 (PMC11957398; doi:10.1117/1.NPh.12.2.025004)
Supplement: Supplementary file 1 [file NPh_012_025004_SD001.pdf]

Supplementary Material

Results for HbR

1. Cortical Response Intensity, Lateralization and Functional Connectivity

Fig S1 shows the statistical analysis results of the metrics calculated based on wavelet analysis. The trends of cortical response intensity, lateralization and functional connectivity were consistent with the results for HbO_2, although there were differences in comparisons within or between groups where significant differences appear.

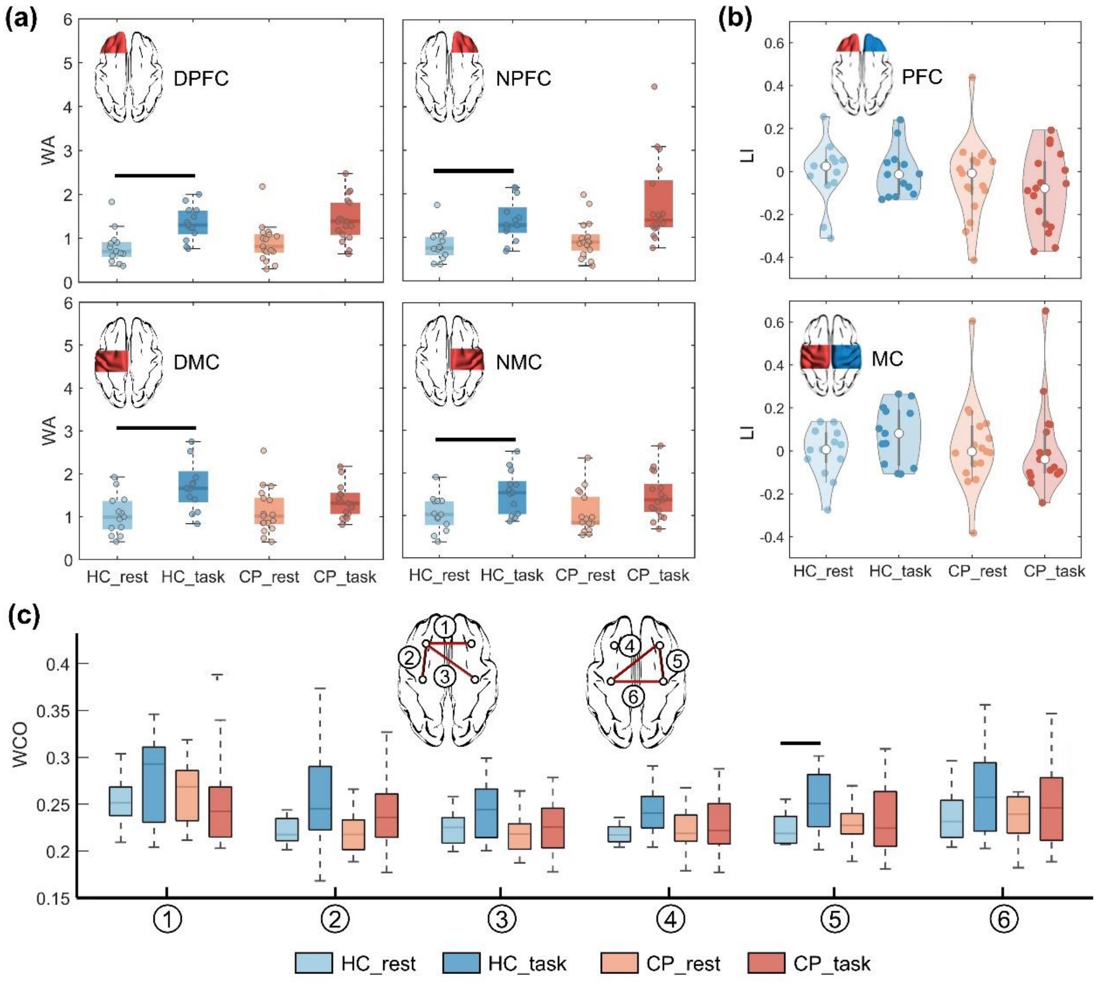

Fig. S1 The WA and LI results for HbR: (a) average WA values of brain region and (b) LI values of the PFC and MC. (c) The WCO between brain regions under different tasks for HbR. A horizontal black line indicates that $p < 0.0125$.

2. *The Graph Theory Parameters*

In graph theory parameters (see Fig S2), the clustering coefficient ($p = 0.008$) and transitivity efficiency ($p = 0.006$) in the HC group were found to be significantly greater during walking than at rest, while the modularity was lower than at rest ($p = 0.008$). In fact, the CP group also exhibited the same trend in these parameter changes (although without significant differences). Although no significant differences were found in global efficiency and local efficiency, similar to HbO_2, the resting state global efficiency in the CP group was higher than in the HC group, and the local efficiency was lower than in the HC group. This also reflects the tendency towards more randomized brain network in children with CP.

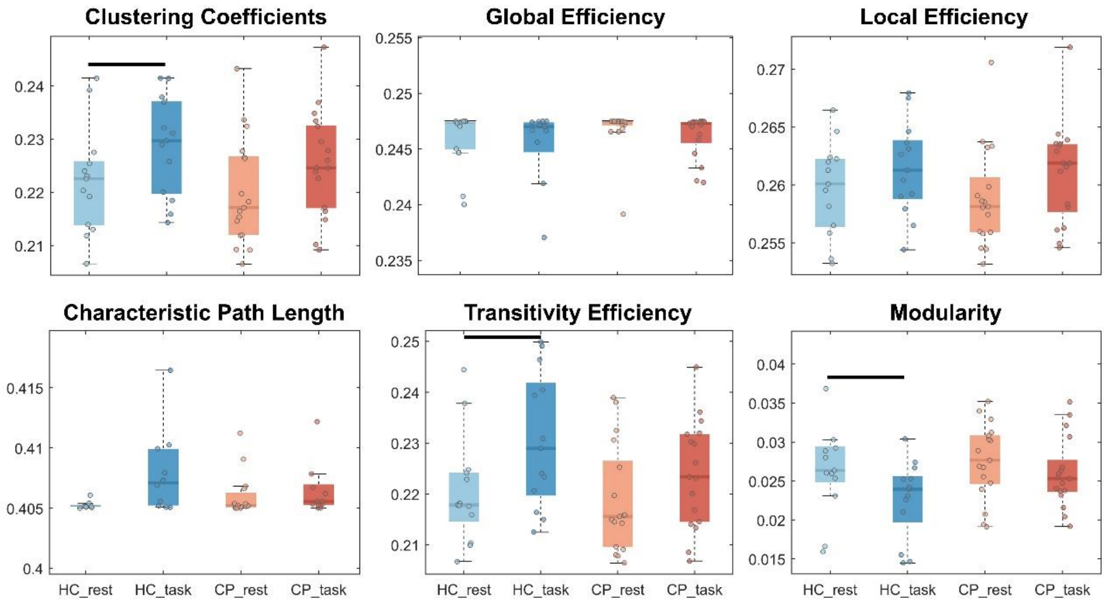

Fig. S2 Results of graph theory analysis for HbR. A horizontal black line indicates that $p < 0.0125$.

3. Effective Network Analysis

The differences in the degree of brain information output and outflow intensity between the two groups of children during rest and task states were comparatively analyzed, as shown in Fig S3. Consistent with the HbO_2 results, during the walking task, the HC group showed decreases in information outflow from the prefrontal cortex (out degree: $p = 0.003$; dPTE value: $p = 0.008$) and an increase in information outflow from the MC.

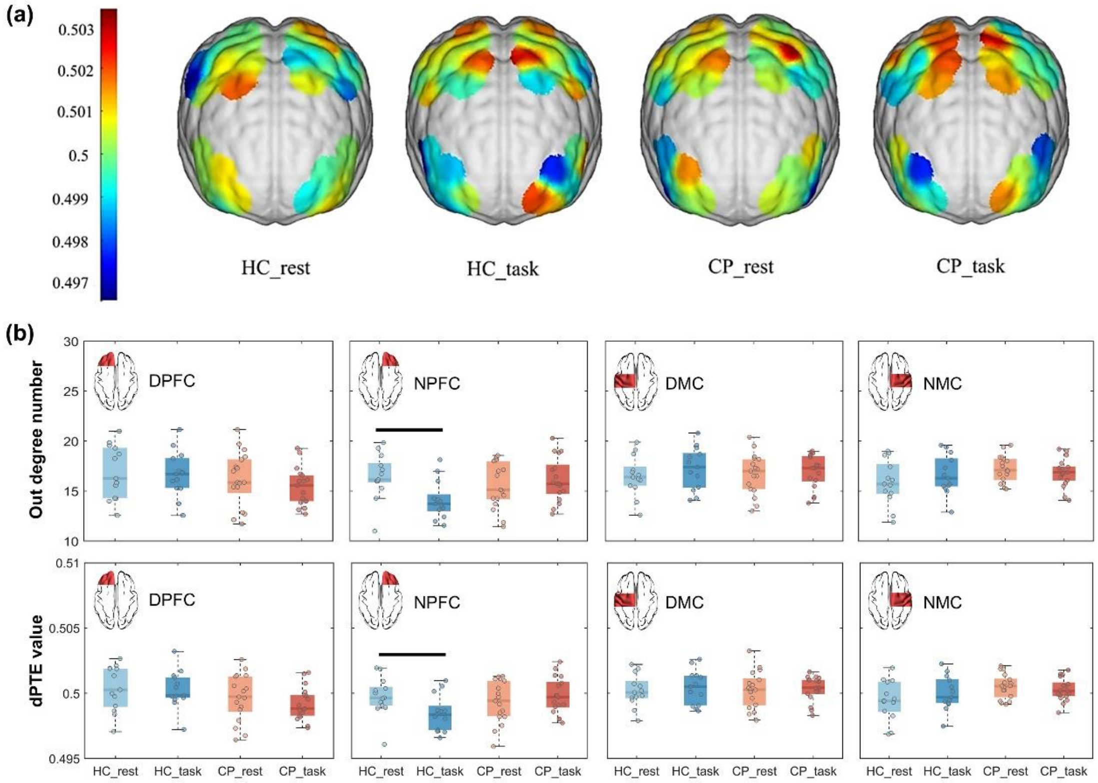

Fig. S3 Results of effective network analysis: (a) average brain information outflow intensity, (b) out-degree number and information outflow intensity of four brain regions. A horizontal black line indicates that $p < 0.0125$.

4. Dynamic Functional Connectivity State

Through cluster analysis, four functional connectivity states of the HbR signal were identified, as shown in Fig S4. Corresponding to the HbO results, among the four states, there was a globally low connectivity state (state 1), a globally high connectivity state (state 4), and two states with high connectivity in local brain regions (state 2 and state 3). State 2 was characterized mainly by high connectivity in the PFC, while state 3 was characterized mainly by high connectivity in the PFC and DMC.

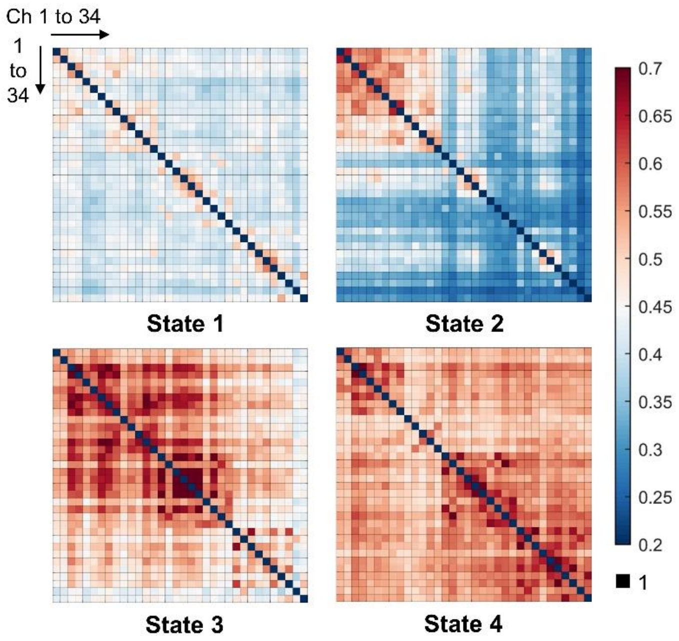

Fig. S4 Four functional connectivity states of the HbR signal.
